# Exploring the Sensorimotor System's Role in Emotional Authenticity Discrimination: A ccPAS Study

**DOI:** 10.1111/nyas.70390

**Published:** 2026-09-03

**Authors:** Thomas Quettier, Fausto Caruana, Cristina Scarpazza, Sara Borgomaneri

**Affiliations:** ^1^ Center for Studies and Research in Cognitive Neuroscience, Department of Psychology “Renzo Canestrari” Cesena Campus, Alma Mater Studiorum Università Di Bologna Cesena Italy; ^2^ Institute of Neuroscience National Research Council of Italy (CNR) Parma Italy; ^3^ Department of General Psychology University of Padua Padua Italy; ^4^ IRCCS San Camillo Hospital Venice Italy

**Keywords:** cortico‐cortical paired associative stimulation, emotional authenticity discrimination, emotional facial expressions, sensorimotor simulation, transcranial magnetic stimulation

## Abstract

Emotional authenticity discrimination (EAD) is key for social interactions. The social cognition hypothesis suggests that emotion recognition relies on a dynamic visuo‐motor interaction. However, the direction and role of this interaction in EAD are still unclear. Here, we investigate the impact of cortico‐cortical paired associative stimulation (ccPAS) on the functional connectivity within the visuomotor networks involved in EAD of facial expressions. Sixty‐four participants were randomly assigned to different ccPAS conditions targeting the backward and forward connectivity between the right inferior frontal gyrus (IFG) and the right posterior superior temporal sulcus (pSTS; IFG–pSTS, pSTS–IFG), and between the IFG and the right primary motor cortex (M1; IFG–M1, M1–IFG). The results revealed improvements in EAD selectively following the pSTS–IFG and the IFG–M1 modulation, across all emotional expressions. Interestingly, a selective increase for genuine emotions was found after IFG–M1 stimulation, suggesting a specific improvement to ecologically valid and socially relevant stimuli. These findings suggest that selectively enhancing the visual‐to‐motor and premotor‐to‐motor pathways facilitates EAD. In contrast, motor‐to‐visual feedback appears to play a negligible role. This study contributes to understanding the neural mechanisms underlying EAD and highlights the potential role of the ccPAS protocol in improving social cognitive abilities, with implications for developing clinical interventions.

## 1 Introduction

People accurately recognize facial emotional expressions [1] but struggle to assess their authenticity [2, 3, 4]. Indeed, altered emotion processing has been observed across several clinical populations, including individuals with autism [5], schizophrenia [6], and psychopathy [7, 8]. Thus, understanding the neural substrates of emotional authenticity discrimination (EAD) may provide important insights into the dysfunctional neural networks underlying these clinical impairments. However, to date, very few studies have been conducted to investigate the neural bases of such human ability. One of the pivotal hypotheses in social cognition posits that the recognition of emotional expressions involves a dynamic interaction between the visual and the motor/premotor systems [9, 10, 11, 12, 13]. While this model has garnered substantial support at a broad level, numerous unresolved queries persist regarding the detailed mechanisms underlying its implementation. Among these, a key issue concerns the directionality of information flow—whether it proceeds from the visual to the motor system, the reverse, or through a more complex bidirectional exchange. Understanding others’ emotions requires the observer to visually analyze and integrate multiple static and dynamic features of facial expressions [4, 14, 15, 16]. A key brain region responsible for the visual processing of facial expressions is the posterior section of the superior temporal sulcus (pSTS) [17, 18, 19, 20, 21]. Notably, transcranial magnetic stimulation (TMS) studies have highlighted the pSTS as crucial for emotion recognition and empathic accuracy (EA) tasks [22, 23, 24, 25]. In addition to visual cortices, observing emotional motor behaviors, such as facial expressions, activates motor (i.e., the primary motor cortex—M1), somatosensory (primary somatosensory cortex—SI), and premotor regions such as the inferior frontal gyrus (IFG) responsible for controlling and sensing facial movements [9, 26, 27, 28, 29, 30]. These visual and motor‐shared activations have led to sensorimotor simulationist models, proposing that perceiving facial expressions is at least partially rooted in the same neural circuits involved in executing and sensing facial movements [10, 11, 13, 31, 32]. Further TMS research supports the essential role of the sensorimotor network in smile recognition [33] and smile authenticity discrimination [12], with functional magnetic resonance imaging (fMRI) studies additionally demonstrating the activation of the IFG to play a key role in mediating emotional responses [34]. Although the role of sensorimotor and visual areas in emotion recognition has been established (for a recent activation likelihood estimation meta‐analysis, see [35]), the neural dynamics subtending EAD are still obscure. Several neurobiological models of emotion processing have been proposed [13, 36, 37, 38, 39], yet these have been based almost exclusively on functional activation and so lack a refined understanding of the connectivity between implicated regions. The coordination of processing stages in different brain regions necessary for an understanding of others’ emotions is well delineated by a theoretical model proposed by Wood et al. [13], which considers the recognition of others’ emotions as a complex process involving the parallel activation of two different systems, one for the visual analysis of faces and facial expressions and a second one for sensorimotor simulation of facial expressions. A crucial aspect of Wood's model is that the brain regions devoted to the simulation mechanism may send information back to visual areas to influence the quality of the representation of facial expressions. However, there is no direct and causal evidence of this fascinating aspect of Wood's model. Connections from STS to IFG have been found in humans [40, 41, 42, 43], also supported by functional connectivity studies on face perception [44] and diffusion tensor imaging results [45], providing the substrate for recurrent signal exchange rather than a simple feedforward visual‐to‐motor cascade [46, 47, 48, 49].

Thus, based on this connectivity profile, common assumptions of the models of the sensorimotor simulation are that visual information enters via the STS, visual information is projected from the STS to the IFG, and motor information is transferred from IFG to STS, closing the visuo‐motor loop. Depending on the modeling framework, the STS and IFG are interpreted as recurrent neural networks [50], implementing a feedforward model from STS to IFG (visual‐to‐motor) and a feedback model from IFG to STS (motor‐to‐visual) [51]. However, it is not clear whether feedforward (i.e., visual‐to‐motor) and feedback (i.e., motor‐to‐visual) connections are critically involved in EAD. Different hypotheses have been formulated to answer this critical question. Wood's model suggests that sensorimotor feedback is critical for refining perception by modulating visual analysis through motor‐derived predictions. According to Wood et al. [13], expression recognition involves an automatic sensorimotor simulation process in which the observer may covertly mimic the observed expression. According to this view, perceiving another person's facial expression activates corresponding sensorimotor representations in the observer, and the resulting bodily or motor feedback contributes to emotion recognition and understanding. This process recruits motor and premotor regions, generating efference copies that anticipate sensory consequences. These predictions are then relayed back to visual areas, where they enhance perceptual clarity and sensitivity to facial expressions. Based on this idea, both feedforward and feedback connections are critical for emotion recognition, and this could be independent of the authenticity of the facial expression.

On the other hand, based on an affordance‐driven perspective, social actions are relevant cues that directly prepare, facilitate, or inhibit specific behavioral responses in the observer [52, 53]. From this perspective, the processing of emotional expressions is primarily linked to their action relevance rather than to the simulation of the observed motor state [54, 55]. Importantly, whereas the sensorimotor simulation account predicts activation of face‐related motor representations, the affordance‐driven perspective may recruit broader motor systems involved in preparing adaptive responses to socially relevant signals. Moreover, while the sensorimotor feedback account focuses on how motor simulation supports emotion recognition, the affordance‐driven perspective highlights how emotional stimuli influence action preparation and control by signaling opportunities or requirements for adaptive behavior. These perspectives are not necessarily mutually exclusive, but they differ in the functional role attributed to motor processes during emotion perception. In this context, the affordance‐driven perspective argues that M1 activation occurs as a reaction to the observed genuine facial expression, reflecting automatic motor resonance mechanisms [48, 56], which should facilitate social synchronization and emotional contagion [57]. In support of this notion, behavioral studies have reported that observing a genuine facial expression triggers an automatic motor response [56, 57] and neuroimaging evidence has demonstrated increased sensorimotor activation in response to genuine expressions compared to posed ones [34, 58], in line with the idea that genuine expressions evoke stronger motor simulation [59, 60]. Specifically, such a perspective supposes the visual input reaches the IFG, which then projects to M1, triggering an automatic motor response.

In line with this idea, TMS studies showed that the IFG differentiates between genuine and posed expressions [12], while the posterior visual cortices seem to be recruited only in low‐performing participants and when more subtle tasks need to be performed (i.e., judging the intensity of emotional expressions [22]). Based on such a hypothesis, only the sensorimotor simulation within the motor and premotor system should play a crucial role in EAD, and the same neural circuits should be particularly relevant for genuine emotion discrimination.

To test these possibilities, we implemented an innovative TMS paradigm, the cortico‐cortical paired associative stimulation (ccPAS), to modulate the functional connectivity between premotor and motor regions (i.e., IFG and M1) and visual‐related regions (i.e., STS). The ccPAS technique employs dual‐coil TMS to influence the synaptic efficiency of cortico‐cortical connections [61, 62, 63, 64]. This protocol utilizes two focal coils to target interconnected cortical regions, promoting Hebbian spike‐timing‐dependent plasticity between them [65]. This protocol has shown promise in enhancing the perception of emotional facial expressions by strengthening connectivity from the STS to visual cortices (V1/V2) [61], offering preliminary support for the crucial role of feedback connections in emotion recognition. Here, we aimed to investigate whether strengthening feedforward and feedback connectivity between the motor and visual systems could improve EAD. According to the simulation model, enhanced EAD should be associated with increased connectivity in both feedforward and feedback pathways, irrespective of whether facial expressions are genuine or posed. By contrast, the affordance‐driven account predicts selective modulation of sensorimotor connectivity underlying EAD, with effects expected to be particularly pronounced during the discrimination of genuine emotional expressions.

Indeed, here we targeted the hand‐related M1 region to investigate whether M1 activation can reflect a more general motor involvement and not only effector‐specific, as has been suggested by previous TMS studies [66, 67, 68].

Moreover, here we tried to overcome the limitation of most previous studies on emotion recognition, that is, the use of posed emotional stimuli (deliberately produced without reflecting the felt emotion) [69, 70]. Some datasets, including both genuine and posed emotions, are now available for research (for a review, see [71]). Still, they have drawbacks (e.g., including only happiness, using only 2D images, and the absence of validation), limiting their usability in clinical and research settings. Our approach of combining ccPAS with a complex socially relevant task, such as EAD, with highly ecological dynamic stimuli depicting different emotions taken from the Padova Emotional Dataset of Facial Expressions (PEDFE) dataset [72] has the potential to provide causal evidence for the involvement and malleability of the networks crucial in EAD, offering further support for sensorimotor simulation models.

## 2 Materials and Methods

### 2.1 Participants

A total of 64 healthy young adults were involved in the study. Participants (average age 23.60 ± 2.14 years) were randomly assigned to one of four ccPAS conditions (IFG–pSTS, pSTS–IFG, IFG–M1, M1–IFG). The four groups were matched for age (*F*_3,60_ = 1.42, *p* = 0.25; *d* = 0.07) and gender (*χ* ^2^_3_ = 3.61, *p* = 0.31). Participants were recruited through printed and electronic advertisements displayed on noticeboards at various University of Bologna sites, as well as through word of mouth. No participant was tested in more than one condition. All participants were right‐handed according to a standard handedness inventory [73], had normal or corrected‐to‐normal visual acuity in both eyes, and were naive to the purposes of the experiment. None had neurological, psychiatric, or medical problems or any contraindications to TMS [74]. Participants provided written informed consent. The procedures were approved by the Bioethics Committee at the University of Bologna and were carried out in accordance with the ethical standards of the Declaration of Helsinki. This study complies with the PECANS guidelines [75]. No discomfort or adverse effects of TMS were reported or noticed during the experimental sessions. The sample size for this experiment was chosen based on prior TMS ccPAS on motion perception [76, 77], and STS–rTMS on emotion perception [22], all showing large effect sizes (Cohen's *d* = 1.10). Using G*Power 3 software [78] with power (1−β) = 0.95 and α = 0.05, we estimated that a sample of 16 participants would be sufficient to show baseline versus post‐ccPAS differences in the experimental groups. At the end of all the experimental phases, participants completed the 20‐item Toronto Alexithymia Scale (TAS‐20 [79]) and the IRI questionnaire [80]. The TAS‐20 is a three‐dimensional self‐reported questionnaire that measures participants’ levels of alexithymia. This self‐report instrument has been demonstrated to have good psychometric properties: internal consistency, Cronbach alpha = 0.81; test–retest reliability *r* = 0.86 [81]. The IRI is a 28‐item self‐report survey that consists of four subscales, namely, perspective taking (PT, which assesses the tendency to spontaneously imagine and assume the cognitive perspective of another person), fantasy scale (FS, which assesses the tendency to imaginatively transpose oneself into fictional situations), empathic concern (EC, which assesses the tendency to feel sympathy and compassion for others in need), and personal distress (PD, which assesses the extent to which an individual feels distress in emotional interpersonal contexts). PT and FS assess cognitive components of empathy, while EC and PD correspond to other‐oriented empathy reactions and self‐oriented emotional distress, respectively [80].

### 2.2 Stimuli

Stimuli consisted of 48 dynamic movies (2 s, 43 frames) presented centrally on a 13‐in. monitor (resolution: 854 × 480; refresh rate: 60 Hz), subtending a visual angle of 17° × 9°. We extracted video clips from the PEDFE database [72].

### 2.3 Stimuli Validation

A total of 65 stimuli were initially selected from the PEDFE dataset: 33 genuine and 32 posed expressions. The genuine condition included 11 stimuli for each emotion category (anger, fear, and happiness), while the posed condition included 11 anger, 10 fear, and 11 happiness stimuli. To validate our subset of stimuli, an independent sample of 30 participants (16 female; mean age 23.2 ± 3 years), not involved in the main experiment, completed the EAD task, in which participants were required to discriminate whether each expression was genuine or posed, and a forced‐choice emotion‐recognition task. The final set comprises 48 stimuli, 24 genuine and 24 posed expressions, with each emotion category (anger, fear, and happiness) represented by eight stimuli in both the genuine and posed conditions. Using this subset of stimuli, participants were able to discriminate emotion authenticity above chance (mean *d*′ = 1.15 ± 0.80) and were able to correctly identify the emotional stimuli (95.4% ± 4.5% correct). Finally, participants showed comparable authenticity sensitivity across the three emotions (*F*_1.93, 55.87_ = 3.02, *p* > 0.05).

### 2.4 Experimental Procedure

The entire experiment was programmed using Psychopy v.2022.1.4 and performed on a screen placed at 70 cm from the participant, with a resolution of 1920 × 1080 and a refresh rate of 60 Hz. Participants were initially presented with task instructions. Each trial started with a black screen (2 s) followed by the video clip, each lasting 2 s. For each video, the participant was asked to perform the EAD task. Following their response, participants were asked to rate authenticity on a Likert scale ranging from −7 to 7, where −7 indicated no authenticity at all, and 7 indicated maximum authenticity. A slider was presented on the screen, and participants used the mouse to click on the desired position along the slider to indicate their rating. Thus, whereas the EAD task required a categorical discrimination decision under forced‐choice conditions, the EA task required participants to rate the authenticity, reflecting a more nuanced and subjective evaluation. Because the continuous scale included a neutral midpoint (0), participants could express uncertainty or intermediate impressions that were not possible in the binary EAD response.

**FIGURE 1:**
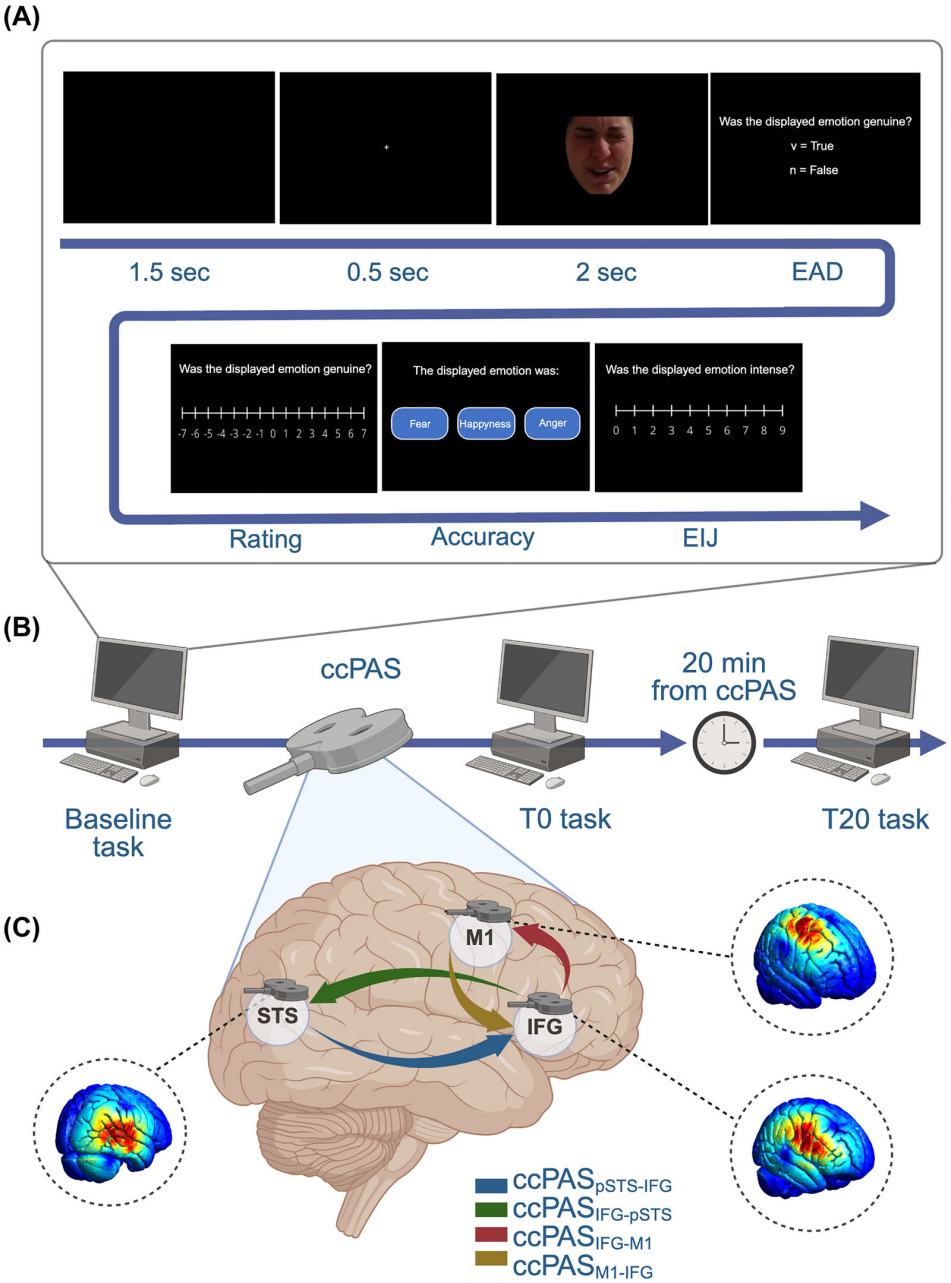
(A) Trial sequence. (B) Experimental procedure: EAD and EIJ tasks were performed before the ccPAS administration (baseline), immediately after the ccPAS administration (T0), and 20 min after the ccPAS protocol (T20). (C) Targeted brain regions for ccPAS and computational simulations (dashed circles) of the estimated electric field distribution resulting from ccPAS application. The volumetric spread of the magnetic field simulation was created using SimNIBS v4.0.1. Abbreviations: ccPAS, cortico‐cortical paired associative stimulation; EAD, emotional authenticity discrimination; EIJ, emotion intensity judgment; IFG, inferior frontal gyrus; M1, primary motor cortex; pSTS, posterior superior temporal sulcus; TMS, transcranial magnetic stimulation (https://BioRender.com).

Finally, we additionally ensured that participants were able to discriminate the emotion displayed in each video clip (i.e., forced choice, mean overall performance was 97 ± 18%). Finally, participants were also asked to perform an emotion intensity judgment (EIJ) task, in which they had to rate the intensity of the emotional expression on a Likert scale from 0 to 9 (0 = not at all intense emotion; 9 = very intense emotion). As for authenticity rating, participants used a slider to indicate their rating. For each participant, an EA score was calculated as the correlation between the participant's ratings and the actors’ self‐reported emotion intensity ratings from the emotion elicitation recorded in the dataset video [22, 82, 83, 84]. EA scores were computed separately for each TMS group, session, and emotion, considering only genuine elicitation (Figure 1A). The behavioral task was presented to participants three times: before the ccPAS protocol (baseline), immediately after the ccPAS (T0), and 20 min after the ccPAS administration (T20) (Figure 1B).

The ccPAS protocols were delivered with a Magstim BiStim2 machine (Magstim Company, UK) via two 50 mm figure‐of‐eight coils placed over the right pSTS and V1/V2. Ninety pairs of stimuli were continuously delivered at a rate of 0.1 Hz for ∼15 min [61, 63, 76], with each pair of stimuli consisting of two monophasic transcranial magnetic pulses. The pulses were triggered remotely using a computer that controlled both stimulators (https://github.com/Merluin/TMS‐TouchPad‐P4R4 [85]) TMS intensity was set to 60% of the maximum stimulator output for the pSTS–IFG and the pSTS–IFG groups [61, 86], while for the IFG–M1 and the M1–IFG groups, the resting motor threshold (rMT) was estimated in a preliminary phase of the experiment using standard procedures [87]. Motor‐evoked potentials (MEPs) induced by stimulation of the right motor cortex were recorded from the left first dorsal interosseous (FDI) using a Biopac MP‐35. Electromyography signals were band‐pass filtered (30–500 Hz) and digitized at a sampling rate of 5 kHz. Pairs of Ag–AgCl surface electrodes were placed in a belly tendon montage with a ground electrode on the wrist. The intersection of the coil was placed tangentially to the scalp with the handle pointing backward and laterally at a 45° angle away from the midline. The optimal scalp position for inducing MEPs from the left FDI was first localized, and the rMT was determined from that position. The rMT was defined as the minimal intensity of stimulator output that produced MEPs with an amplitude of at least 50 µV in the FDI with 50% probability [88]. The ccPAS protocol was manipulated in different groups of participants (Figure 1C).

#### 2.4.1 pSTS–IFG

In each TMS pair, the first pulse was delivered to pSTS and followed by a second pulse delivered to IFG with an interstimulus interval (ISI) of 40 ms. This timing was found to be critical in our previous TMS–electroencephalography (EEG) study to induce convergent activation of the dorsal pathway via stimulation of pSTS and IFG. Since theories of sensorimotor simulation claim that expression recognition is mediated by the recruitment of a visuomotor pathway, the rationale for this condition is to test whether an enhancement of the pathway from visual (pSTS) to motor (IFG) areas would produce an improvement in emotional authenticity discrimination abilities.

#### 2.4.2 IFG–pSTS

In this condition, we reversed the direction of the associative pulses: the first pulse was delivered to the IFG, followed by a second pulse to the pSTS, maintaining the same ISI as in the experimental condition (40 ms). Hence, it helped investigate whether any observed effects following the STS‐IFG condition were the result of enforced feedforward connections (from pSTS to IFG) and did not arise when the order of the pulses was reversed. This protocol was, therefore, designed to disentangle between feedforward projection from the visual to the sensorimotor systems and reentrant connections from the sensorimotor system to the visual one.

#### 2.4.3 IFG–M1

The pulse intensity over right M1 was set at 120% of the rMT, and stimulation over M1 was preceded with an ISI of 6 ms by a pulse delivered to the IFG set at 90% of the rMT [89]. This condition was aimed at studying a second step of the sensorimotor simulation hypothesis, namely, whether the sensorimotor simulation mechanism stops at the level of the IFG or vice versa, and reaches to M1. In fact, given the absence of direct connections between the visual system and M1, our experimental hypothesis was that IFG may act as a conduit for M1.

#### 2.4.4 M1–IFG

In this condition, we reversed the direction of the associative pulses: the first pulse was delivered to M1, followed by a second pulse to IFG, using the same ISI as in the experimental condition (6 ms). This condition was essentially a control test for direction‐dependent effects, verifying whether any observed effects in the experimental condition (IFG–M1) were specifically attributable to the specific connectivity direction (from IFG to M1) and not replicated when the order of the pulses was reversed.

### 2.5 Neuronavigation

In all experiments, the pSTS and IFG sites were individually targeted using image‐guided neuronavigation. The positions of the two coils were identified on each participant's scalp using the SofTaxic Navigator System (Electro Medical Systems) as in prior research [61, 63, 73, 90]. Skull landmarks (nasion, inion, and 2 preauricular points) and ∼100 points providing a uniform representation of the scalp were digitized by means of a Polaris Vicra digitizer (Northern Digital). An individual estimated magnetic resonance image (MRI) was obtained for each subject through a 3D warping procedure, fitting a high‐resolution MRI template with the participant's scalp model and craniometric points. This procedure has been proven to ensure a global localization accuracy of roughly 5 mm [91]. Stimulation sites were identified in Talairach space on the basis of previous fMRI and TMS studies. When necessary, MNI coordinates were converted into Talairach space using GingerALE v. 2.3.1. The pSTS was localized in the right hemisphere at the coordinates *x* = 53, *y* = −49, *z* = 10, estimated by averaging subject‐weighted coordinates identified in a meta‐analysis [92] during emotion evaluation (75 experiments, 1742 participants) and passive observation of emotional facial expressions (20 experiments, 411 participants). The pSTS site is confirmed by brain imaging meta‐analyses on emotional face perception [20, 93] and prior TMS studies [22, 94], falling within the range of interindividual variability of the face‐selective area in the pSTS reported by Sliwinska and Pitcher [25]. The IFG scalp site was localized based on the following Talairach coordinates: *x* = 47, *y* = 8, *z* = 28, which were identified on the basis of previous fMRI meta‐analyses exploring activations associated with the execution and/or observation of facial movements and emotional expressions [12, 22, 95, 96, 97]. This site corresponds to the posterior sector of the IFG (BA44, at the border with the ventral premotor cortex), which plays a role in sensorimotor simulation [98, 99]. Locations of scalp regions identified by neuronavigation (pSTS, IFG) or anatomical methods (M1) were marked with a pen on each participant's head and used to place the ccPAS coil. Then, individual Talairach coordinates corresponding to the projection of the targeted scalp sites onto the surface of the MRI‐constructed stereotaxic template were automatically estimated through the neuronavigation system. These estimated coordinates indicate the most superficial cortical site where ccPAS effects are expected to be maximal. Mean coordinates identified for the pSTS were *x* = 57.29 ± 2.41, *y* = −49.05 ± 3.69, *z* = 14.61 ± 6.38. For IFG, the mean coordinates were *x* = 51.09 ± 6.16, *y* = 7.62 ± 3.46, *z* = 29.32 ± 6.09. Lastly, M1 mean coordinates were *x* = 27.21 ± 10.09, *y* = −11.03 ± 15.33, *z* = 57.39 ± 9.11. Furthermore, SimNIBS v4.0.1 [100] was used to estimate the electric field distribution induced by TMS and for automatic skull segmentation from MR images [101] (Figure 1C).

### 2.6 Data Processing and Analysis

Data preprocessing and metric estimation were carried out using custom R scripts tailored to the experiment. All statistical analyses were performed in R (R Foundation for Statistical Computing, Vienna, Austria), using a combination of packages and custom functions. The analysis of EAD focused on sensitivity (*d*′) and decision bias (*criterion*) derived from signal detection theory. ANOVAs were conducted using the *afex* package v.1.3‐1, with models structured to examine effects of Session (3 levels: baseline, T0, T20), Group (4 levels: pSTS–IFG, IFG–pSTS, IFG–M1, M1–IFG), and Emotion (3 levels: Anger, Fearful, Happiness). For post‐hoc analyses, the *emmeans* package v.1.10.0 was employed, and statistical significance was set at *p* < 0.05, with Bonferroni adjustments applied. Gain analysis was performed on *d* delta (T − baseline) to assess whether changes in sensitivity were significantly different from zero. This analysis provided additional insights into the magnitude and direction of sensitivity improvements following the intervention. Discrimination sensitivity was indexed primarily by *d′*, which integrates hit rate (HR; correct identification of genuine expressions, i.e., signal) and false‐alarm rate (FAR; misclassification of posed expressions as genuine, i.e., noise) into a single criterion‐independent measure. Because *d′* is a composite, a given change in *d′* can arise from a change in HR, FAR, or both. To localize the source of any *d′* effect, HR and FAR were, therefore, analyzed separately using the same ANOVA applied to *d′*. These analyses were exploratory and not intended as an alternative index of discrimination; rather, they indicate whether an effect on *d′* was driven by enhanced detection of genuine expressions (HR), reduced misclassification of posed expressions (FAR), or both. Because HR and FAR are each sensitive to response bias, they are interpreted alongside the criterion estimate so that sensitivity‐driven and bias‐driven contributions can be distinguished. We additionally computed the area under the ROC curve (AUC) from the full −7 to +7 authenticity scale, using each clip's true status as ground truth [102] as a threshold‐free index of discrimination. AUC was estimated per participant, session, and group with the *pROC* package and analyzed using the same repeated‐measures ANOVA as *d′*, with a sensitivity analysis excluding neutral ratings. For the EIJ task, we analyzed differences in EA scores across groups, sessions, and emotions; due to non‐normality distribution, a generalized linear model with a beta distribution was applied. Model comparisons and selections were guided by the Akaike information criterion (AIC) and significance thresholds, using Type III Wald chi‐square tests. Post‐hoc contrasts were performed with the *emmeans* package, with pairwise comparisons adjusted using Bonferroni correction. Descriptive statistics were calculated for emotion recognition accuracy, and group‐wise performance was reported as percentages with standard deviations. To investigate the relationship between changes in the EAD performance and dispositional empathy or alexithymic traits, ANCOVA models were applied. The dependent variable was the difference in EAD performance pre‐ and post‐ccPAS stimulation (i.e., EAD of merged emotions post‐ccPAS minus baseline, referred as ΓEAD). Predictor variables included TAS‐20 scores and IRI subscales (i.e., perspective taking, fantasy, empathic concern, and personal distress), alongside group as a between‐subject factor. The significance threshold was set at *p* < 0.05 for all analyses.

## 3 Results

### 3.1 Emotional Authenticity Discrimination

**FIGURE 2:**
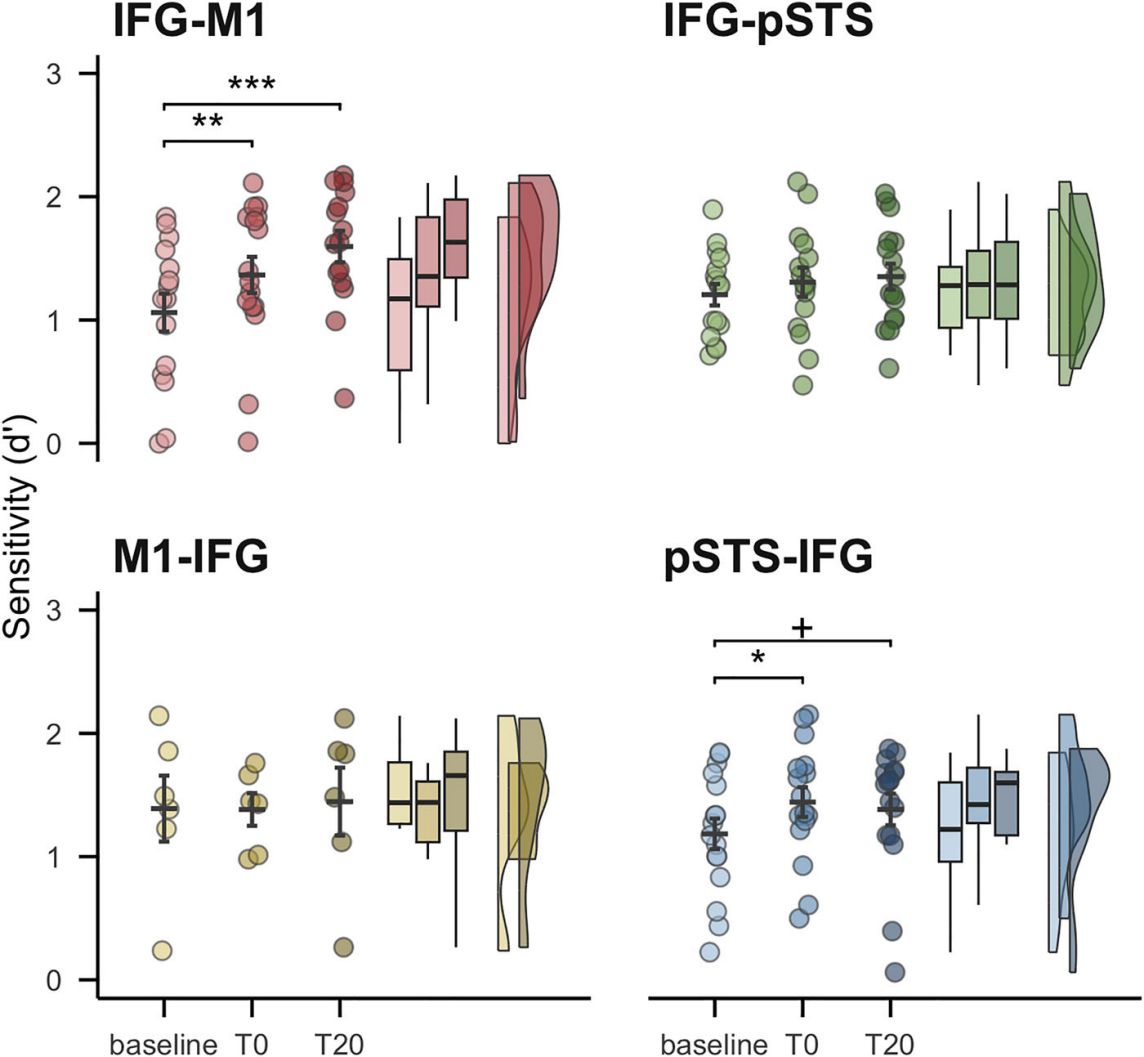
Mean EAD sensitivity (*d′*) across sessions (baseline, T0, T20) and groups. Individual points represent data from individual participants with mean and standard error, box plots summarize the median and interquartile range, and flat violin plots depict the density distribution of the data. Colors represent the different experimental groups: red for IFG–M1, green for IFG–pSTS, yellow for M1–IFG, and blue for pSTS–IFG. + denotes *p* < 0.1; *p < 0.05; **p < 0.01; and ***p < 0.001.

The ANOVA, *d′ ∼ session* (3 levels: baseline, T0, T20) *× group* (4 levels: pSTS–IFG, IFG–pSTS, IFG–M1, M1–IFG) *× emotion* (3 levels: Anger, Fearful, Happiness), indicated significant main effects of session (*F*_1.92, 115.20_ = 14.95, *p* < 0.001), emotion, (*F*_1.86, 111.89_ = 19.83, *p* < 0.001) but not group (*F*_3, 60_ = 1.24, *p* = 0.31). A significant session × group interaction was found (*F_5_*_.76, 115.20_ = 2.48, *p* = 0.02). Post‐hoc analysis revealed that in the IFG–pSTS and M1–IFG groups, no significant differences were found between sessions (all *t* < 1.65; all *p*s > 0.23). Interestingly, significant differences were observed between T0 and baseline (*t*_60_ = −2.51, *p* = 0.04; *t*_60_ = −3.48, *p* < 0.01) in the pSTS–IFG and IFG–M1 groups. Moreover, significant differences were observed between T20 sessions and baseline for the IFG–M1 group (*t*_60_ = −5.87, *p* < 0.001) and marginally for the pSTS–IFG group (*t*_60_ = −2.24, *p* = 0.08; Figure 2). Interestingly, no significant session × emotion × group interaction was found (*F*_11.31, 226.10_ = 0.74, *p* = 0.71), indicating that the differences between sessions and groups were consistent across all emotions.

The ANOVA, criterion ∼ session (3 levels: baseline, T0, T20) *×* group (4 levels: pSTS–IFG, IFG–pSTS, IFG–M1, M1–IFG) *×* emotion (3 levels: anger, fearful, happiness), indicated significant main effects of emotion (*F*_1.86, 111.33_ = 28.36, *p* < 0.001) but not group (*F*_3, 60_ = 0.43, *p* = 0.73) or session (*F*_1.71, 102.68_ = 0.44, *p* = 0.61). No significant interactions were found (all *F*s < 1.45; all *p*s > 0.22).

These results suggest that the ccPAS protocol targeting pSTS–IFG and IFG–M1 connectivity selectively enhances EAD, as reflected by significant improvements in sensitivity (*d'*) across sessions for these groups, indicating increased accuracy in distinguishing between genuine and posed emotions, and was consistent across emotions. In contrast, no significant changes in *d'* were observed in the control groups (IFG–pSTS and M1–IFG). For the bias (criterion), there were no significant effects of session or group, indicating that while sensitivity to authenticity improved, there was no notable shift in participants’ decision bias across any of the groups or sessions (Figure 2 and Table 1).

**TABLE 1:** Emotional authenticity discrimination sensitivity (*d′*) by group and session.

Group	baseline	T0	T20
IFG–M1	1.06 ± 0.59	1.37 ± 0.59	1.60 ± 0.50
IFG–pSTS	1.21 ± 0.35	1.22 ± 0.55	1.35 ± 0.42
M1–IFG	1.39 ± 0.66	1.38 ± 0.32	1.45 ± 0.67
pSTS–IFG	1.18 ± 0.50	1.44 ± 0.48	1.38 ± 0.51

*Note*: Values are mean ± *SD* across participants for each experimental group at baseline, T0, and T20. Higher *d*′ indicates better discrimination of genuine from posed emotional expressions.

Abbreviations: IFG, inferior frontal gyrus; M1, primary motor cortex; pSTS, posterior superior temporal sulcus.

#### 3.1.1 Delta Analysis

**FIGURE 3:**
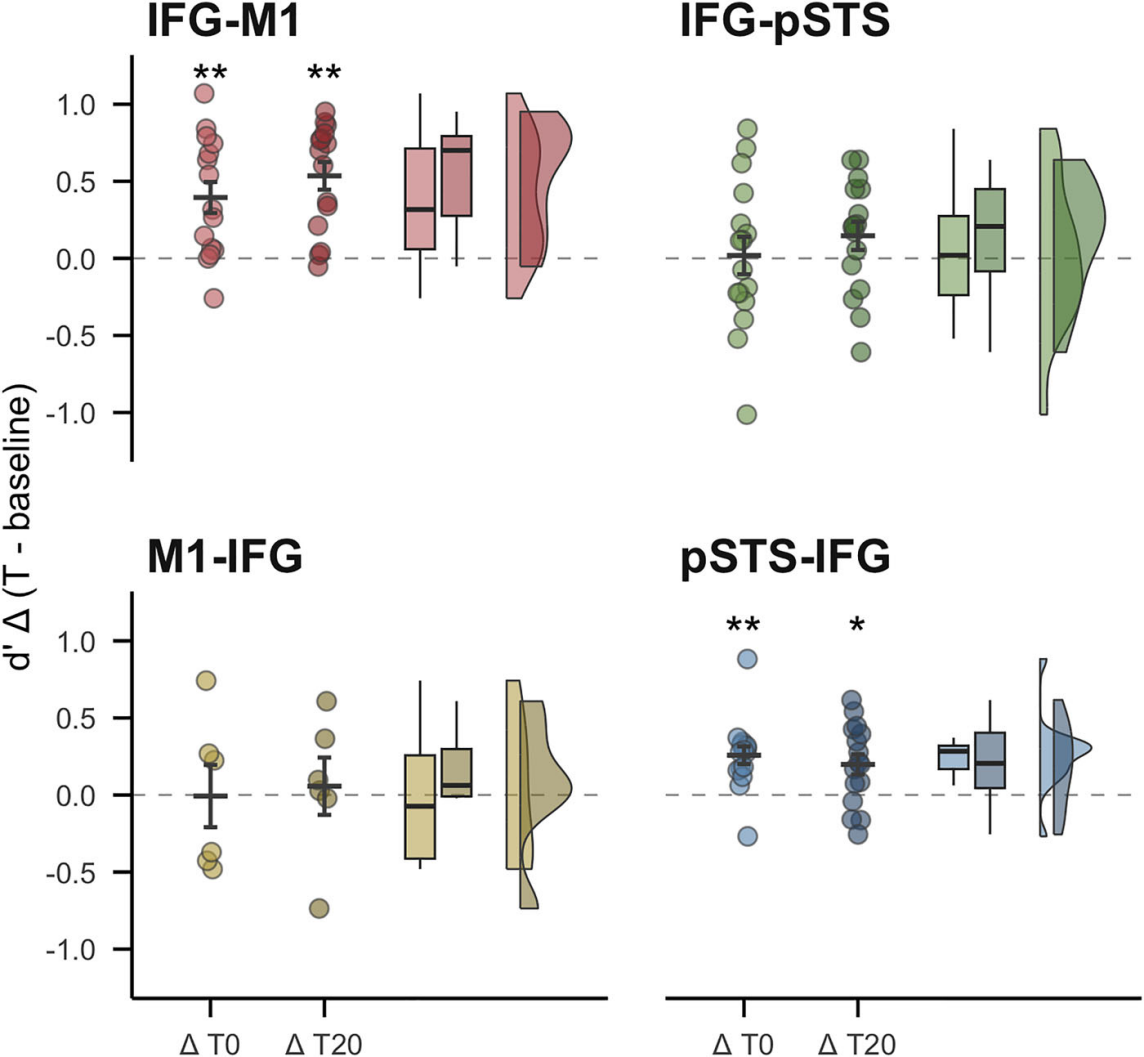
Discrimination sensitivity (*d′*) delta (T − baseline) and groups. Individual points represent data from individual participants with mean and standard error, box plots summarize the median and interquartile range, and flat violin plots depict the density distribution of the data. Colors represent the different experimental groups: red for IFG–M1, green for IFG–pSTS, yellow for M1–IFG, and blue for pSTS–IFG. + denotes *p* < 0.1; *p < 0.05; **p < 0.01; and ***p < 0.001.

To assess the changes in emotional discrimination sensitivity between sessions, we calculated the gain as the difference (Δ) in *d′* from the baseline to each subsequent session (Δ = T − baseline). A one‐sample *t*‐test was performed for each group and session to determine whether the gain was significantly different from zero. For the T0 − baseline comparison, significant positive gains were observed in the pSTS–IFG group (Δ = 0.26 ± 0.62, *t*_47_ = 2.88, *p* = 0.006, CI [0.078, 0.438]) and the IFG–M1 group (Δ = 0.36 ± 0.78, *t*_47_ = 3.18, *p* = 0.003, CI [0.131, 0.584]). No significant gains were detected in the other groups (IFG–pSTS: Δ = 0.02 ± 0.67, *t*_47_ = 0.19, *p* = 0.85, CI [−0.176, 0.213]; M1–IFG: Δ = 0.08 ± 0.81, *t*_47_ = 0.73, *p* = 0.47, CI [−0.150, 0.321]). For the T20 − baseline comparison, a significant positive gain was observed in the IFG–M1 group (Δ = 0.52 ± 0.68, *t*_47_ = 5.31, *p* < 0.001, CI [0.322, 0.716]) and, to a lesser extent, in the pSTS–IFG group (Δ = 0.20 ± 0.62, *t*_47_ = 2.20, *p* = 0.03, CI [0.017, 0.379]). However, gains in the control groups remained nonsignificant (IFG–pSTS: Δ = 0.15 ± 0.70, *t*_47_ = 1.45, *p* = 0.15, CI [−0.057, 0.350]; M1–IFG: Δ = 0.13 ± 0.67, *t*_47_ = 1.31, *p* = 0.19, CI [−0.068, 0.321]). These findings converge with the EAD results, indicating that the ccPAS protocol targeting pSTS–IFG and IFG–M1 connectivity significantly enhanced sensitivity to EAD, as reflected by greater gains in *d′* after ccPAS administration relative to baseline. No significant changes were observed in the other groups, underscoring the specificity of the experimental manipulations (Figure 3).

#### 3.1.2 Rate Analysis

The analysis investigated differences in HR and FAR across groups, sessions, and emotion types in the EAD task. The additional analyses on HR and FAR were not intended as independent tests of discrimination sensitivity, but rather as exploratory control analyses aimed at clarifying the source of any observed changes in *d′*. Specifically, because genuine expressions may possess greater emotional contagion or salience than posed expressions, an experimental manipulation could theoretically affect responses selectively for genuine expressions (signal), selectively for posed expressions (noise), or both. Examining HR and FAR separately, therefore, allowed us to determine whether changes in *d′* reflected: (i) improved detection of genuine expressions; (ii) reduced misclassification of posed expressions; or (iii) a combined effect on both components. This distinction is crucial, as it explores potential effects of the ccPAS stimulation that may not be observable through the *d′* analysis alone. An ANOVA was performed with rate as the dependent variable and rate type (HR vs. FAR), emotion (3 levels: anger, fearful, happiness), session (3 levels: baseline, T0, T20), and group (4 levels: pSTS–IFG, IFG–pSTS, IFG–M1, M1–IFG) as factors. The results revealed a significant main effect of emotion (*F*_1.82,108.97_ = 28.26, *p* < 0.001), indicating that the type of emotion influenced the rate. Additionally, the main effect of rate type (*F*_1,60_ = 453.04, *p* < 0.001) reflected substantial differences between HR and FAR across all conditions. Importantly, a significant interaction between emotion and rate type (*F*_1.90,114.21_ = 18.35, *p* < 0.001) was observed, suggesting that the influence of emotion varied between HR and FAR. Furthermore, a significant rate type × session × group interaction was found (*F*_5.77,115.40_ = 2.81, *p* = 0.02), indicating that changes in HR and FAR over sessions differed across groups. No other significant interactions involving emotion, session, or group were found (all *F*s < 1.65, all *p*s > 0.17).

Post‐hoc analyses revealed that in the IFG–M1 group, HR significantly increased from baseline to T0 (Δ = −0.078, *p* = 0.009) and from baseline to T20 (Δ = −0.111, *p* < 0.001), indicating improved sensitivity to genuine emotions following ccPAS administration. In contrast, the pSTS–IFG group showed no significant HR changes (all *p*s > 0.06). For FAR, no significant reductions were observed in these groups across sessions, suggesting stable performance in rejecting posed emotions (all *t*s < 1.85, all *p*s < 0.21). On the other hand, the IFG–pSTS and M1–IFG groups showed no significant changes in HR or FAR across sessions, indicating no significant effects of the administered ccPAS (all *t*s < 1.25, all *p*s < 0.64).

These findings highlight that the ccPAS protocol targeting IFG–M1 selectively enhanced participants’ sensitivity to genuine emotions (HR), while maintaining stable rejection rates for fake emotions (FAR). The lack of significant changes in the other groups further underscores the specificity of the ccPAS effects. Overall, these results support the hypothesis that ccPAS targeting specific neural pathways enhances the discrimination of genuine emotions, with effects consistent across all tested emotions.

#### 3.1.3 ROC Analysis

To confirm that reducing the graded authenticity ratings to a binary response did not discard discriminative information, we computed a threshold‐free measure (area under the ROC curve, AUC) from the full −7 to +7 scale, using each clip's true status as ground truth [102]. AUC improved significantly across sessions (*F*_1.98, 96.97_ = 5.95, *p* = 0.004; and this effect was still significant excluding neutral ratings; *p* = 0.01), with no group effect (*F*_3, 49_ = 0.13, *p* = 0.94) and no session × group interaction (*F*_5.94, 96.97_ = 1.34, *p* = 0.25). The graded −7 to +7 rating is also a more subjective and variable response than the forced binary judgment, reflecting individual differences in scale use and graded uncertainty that the binary choice removes; this additional response variance can reduce sensitivity to a session × group interaction even when the underlying effect is comparable to that captured by *d′*. Consistent with this, although the interaction was not significant, planned baseline‐versus‐post contrasts were computed. The IFG–M1 group showed a significant improvement already at T0 (baseline to T0: Δ = 0.047, *p* = 0.006), which further increased at T20 (baseline to T20: Δ = 0.068, *p* < 0.001), whereas none of the other groups showed significant effects (all *p*s > 0.06). These findings demonstrated that the IFG–M1 group showed improved discriminative performance, as indicated by higher *d′* and ROC AUC values. There was no effect on performance in the pSTS–IFG group as assessed by ROC analysis of the subsequent graded ratings, even though the ccPAS increased sensitivity in the binary discrimination task (higher *d′*).

### 3.2 Emotion Intensity Judgment

Based on AIC and *p* value, the fit1 model EA ∼ emotion + (1 | subject) provided the best fit. Likelihood ratio tests showed significant improvements in fit for fit1 compared to models with additional predictors (fit0 through fit6). However, the interaction model fit4 (EIJ ∼ session × group + (1 | subject)) was not found to significantly improve model fit (*χ* ^2^_8_ = 5.28, *p* = 0.73), indicating that the inclusion of the interaction term does not provide additional explanatory power for the data. The analysis of deviance table (Type III tests) indicated significant main effects of the factor emotion (*χ* ^2^_2_ = 125.5, *p* < 0.001). Post‐hoc contrasts using estimated marginal means revealed that participants’ EA for happy expressions was not different than EA for fearful expressions (*t*_510_ = 0.91, *p* > 0.37), and anger expressions were significantly different from both happy (*t*_510_ = −9.22, *p* < 0.001) and fearful expressions (*t*_510_ = −10.12, *p* < 0.001). In conclusion, these results indicate that participants had lower EA for angry facial expressions relative to both happy and fearful expressions.

### 3.3 Relation Between Changes in EAD and Dispositional Empathy or Alexithymic Traits

To explore the relationship between improved EAD and personality traits, we conducted an analysis of covariance (ANCOVA), with the alpha criterion set at 0.05. The dependent variable was an index representing the EAD for pooled emotions at baseline compared to those collected after the ccPAS (i.e., ΓEAD). Scores from the TAS‐20 and subscales of the IRI were included as covariates. No significant effects of personality traits were found for either TAS‐20 or the IRI subscales in both pSTS–IFG or IFG–M1 groups (all *F*s < 2.77; all *p*s > 0.13).

## 4 Discussion

A popular hypothesis in cognitive neuroscience [32] suggests that EAD depends on the interaction between temporal and frontal regions and is responsible for visual encoding and sensorimotor simulation, respectively, needed to perform the task. While the concurrent activation of these regions during EAD has been established in several studies [4, 12, 58, 59, 60], the mechanisms underlying the actual implementation of this model remain unclear. In this study, we addressed two key questions. First, we explored whether the ccPAS manipulation would influence EAD by modulating feedforward visual‐to‐premotor information flow or premotor‐to‐visual feedback signals. Second, we examined whether the endpoint of frontal encoding occurs in the IFG or, as suggested by the simulationist model, extends to M1. To investigate this issue, we applied an innovative TMS protocol named ccPAS to modulate the functional connectivity between pSTS and IFG, as well as between IFG and M1, in both feedforward and feedback directions, to test whether strengthening these pathways would facilitate the ability to discriminate between genuine and posed emotional expressions.

Our results demonstrate that selectively enhancing the functional connectivity along specific neural pathways significantly improves EAD. Specifically, stimulation of the pSTS–IFG and IFG–M1 pathways led to significant improvements in EAD, whereas stimulation of the IFG–pSTS and M1–IFG pathways did not yield comparable effects, suggesting the ccPAS‐induced plasticity to be pathway‐specific. Interestingly, these improvements were observed across different emotional expressions (anger, fear, and happiness), suggesting that the mechanisms underlying EAD are not limited to specific affective categories but instead support a more generalized processing system. This result is in line with previous evidence showing the involvement of the STS [23, 24, 25] and the IFG [34, 103] in emotional discrimination tasks without any emotion‐specific effect. Overall, our finding aligns with sensorimotor simulation models, which propose that facial emotion recognition is facilitated by motor resonance mechanisms [13, 31]. The ability to simulate the observed expression likely enhances perceptual discrimination, indicating that these sensorimotor circuits play a fundamental role in refining authenticity perception. However, the fact that an increase in EAD is found when targeting the MI hand representation suggests that EAD does not rely on a strict simulation process, but suggests a more general motor engagement. This effect is in line with previous TMS evidence reporting increased corticospinal excitability in hand representation when observing emotional faces and bodies [66, 67, 68].

Additionally, our statistical analyses revealed that the observed improvements in EAD in the IFG–M1 group were primarily driven by an increase in hit rate for genuine expressions, rather than a decrease in false alarm rate. This specificity suggests that ccPAS in IFG–M1 selectively enhances sensitivity to genuine rather than posed expressions. Thus, in line with the affordance‐driven account, the IFG–M1 connectivity was found to be preferentially involved when genuine expressions need to be processed.

Our results extend previous research demonstrating the critical role of the pSTS in emotion recognition and authenticity discrimination [22, 23]. While previous studies primarily focused on the disruptive effects of pSTS inhibition, our findings provide novel evidence that enhancing the functional connectivity between pSTS and IFG can causally improve EAD. This suggests that the pSTS–IFG pathway supports a feedforward processing mechanism, in which visual information from the pSTS is integrated with motor representations in the IFG, refining the perceptual interpretation of emotional expressions. In line with Duchaine and Yovel's model of face processing [36], which emphasizes the role of pSTS and IFG in dynamic facial recognition, our data reinforced the idea that these regions act as a hub for integrating visual and motor information in social perception. Beyond the pSTS–IFG pathway, our findings indicate that the IFG–M1 pathway also contributes significantly to EAD. The transition from pSTS–IFG to IFG–M1 involvement suggests a hierarchical processing structure, whereby motor representations generated in IFG are further refined in M1. This result adds novel support to the sensorimotor simulation hypothesis. The mere recruitment of the IFG as the receptacle of visual outputs from pSTS, in fact, is not sufficient to support the simulationist model, as its reactivity to emotional displays has been frequently explained in terms of cognitive control, decision‐making, self‐perspective inhibition, and working memory [92, 104]. Furthermore, Del Vecchio et al. [105] recently highlighted that the reactivity of the anterior part of IFG to emotional expressions can be of an attentional nature, due to the fact that the IFG is part of the ventral attentional systems specialized for the detection of behaviorally relevant stimuli [106, 107, 108]. Our results instead show that ccPAS induces improvements in the IFG–M1 condition, suggesting that IFG is a bypassing station of connectivity between the temporal and frontal lobes and revealing M1 as the endpoint of the simulation process. Considering that M1 has been traditionally associated with overt motor execution rather than social perception [109], this finding supports the simulationist model, suggesting that EAD may require the activation of primary motor areas.

A particularly intriguing aspect of our findings is the directionality‐specificity in the connectivity effects. While strengthening pSTS–IFG and IFG–M1 pathways enhanced EAD, the reversed stimulation order (IFG–pSTS and M1–IFG) did not yield comparable improvements. This asymmetry suggests that the flow of information in emotional authenticity processing is predominantly feedforward. If motor engagement were purely assisting visual analysis, one might expect reciprocal feedback to pSTS to be equally beneficial. Instead, our results indicate that IFG functions as a conduit for transmitting information to M1, reinforcing the idea that motor contributions to facial emotion recognition do not merely refine perception but actively contribute to action preparation in response to social cues. However, our findings do not rule out a potential functional contribution of feedback connections, which has been found to be particularly important when stimuli are degraded, noisy, or otherwise ambiguous [110, 111]. Thus, it is possible to speculate on the involvement of motor‐to‐visual feedback projections making the stimuli noisier or when different ISIs are employed in the ccPAS protocol.

Overall, our findings suggested that the pSTS–IFG pathway appears to refine perceptual representations by integrating visual and motor information, enhancing the observer's ability to detect authenticity cues. On the other hand, the IFG–M1 pathway is more directly involved in translating these perceptual cues into motor responses, facilitating covert simulation and action preparation in response to genuine expressions. This functional division suggests that while pSTS–IFG primarily optimizes the sensory discrimination process, IFG–M1 serves as a bridge to motor engagement, reinforcing the link between social perception and adaptive behavioral responses. These distinct contributions can be interpreted through two complementary frameworks. First, the sensorimotor feedback hypothesis suggests that facial emotion recognition involves refining visual representations through motor‐derived predictions. In this framework, visual input processed in the pSTS is projected to the IFG, where it is integrated with motor simulation processes, ultimately enhancing perceptual EAD [13, 31]. The pSTS–IFG pathway appears to function as a mechanism for improving perceptual clarity by utilizing predictive models based on prior sensorimotor experiences.

An alternative but complementary explanation is the affordance‐driven hypothesis, which posits that IFG–M1 functional connectivity enhancements facilitate action preparation in response to genuine expressions. Genuine facial expressions serve as strong social affordances, automatically engaging motor circuits in anticipation of an appropriate social response [33, 59]. The fact that strengthening the IFG–M1 pathway enhances EAD is compatible with the hypothesis that sensorimotor integration extends beyond perceptual refinement and includes preparatory motor engagement. Importantly, these two hypotheses are not mutually exclusive; rather, they reflect different stages of processing. The findings of this study contribute to the theoretical understanding of EAD by providing causal evidence for the role of sensorimotor networks in this process. These results reinforce sensorimotor simulation and embodied cognition models of emotion perception [31, 32] and emphasize the importance of distinct pathways in facial expression recognition.

### 4.1 Limitations and Future Perspectives

Our results expand recent findings from animal studies that directly examine the circuits implicated in emotional discrimination and emotional contagion [112]. Importantly, however, although our results reveal the crucial importance of specific cortical networks, we cannot exclude other circuits such as cortico‐thalamo‐cortical loops [113, 114] or structures like the retrosplenial cortex [112] that could also contribute to EAD. Crucially, although our results suggest that enhanced IFG–M1 connectivity improves EAD but that boosting M1–IFG connectivity does not, it is possible that the latter pathway could be preferentially modulated using different tasks, stimulation timing, or effector‐specific representations (e.g., hand vs. orofacial). This would help rule out the possibility that M1–IFG stimulation interferes with IFG processing rather than simply failing to enhance it, thereby limiting the interpretability of the null effect observed for the feedback connection. Moreover, the increase in EAD observed in the STS–IFG group at the 20‐min follow‐up reached only marginal significance, suggesting that the effect may be less robust or stable over time. However, TMS effects can be bidirectional—retrograde as well as anterograde—depending on the underlying cortico‐cortical architecture, excitatory versus inhibitory pathways, and individual variability. Thus, additional studies are required to further investigate the duration of the effects. Moreover, future studies should explore the neural mechanisms underlying these effects by combining ccPAS with TMS–EEG or fMRI to track real‐time connectivity changes during ccPAS stimulation. Moreover, it would be interesting to systematically assess the existence of gender‐specific effects or the use of other emotional stimuli (i.e., emotional body) to test the generalizability of our results.

Beyond theoretical implications, these findings have potential clinical relevance. Given that deficits in social cognition are common in neurological and neuropsychiatric disorders where disruptions of sensorimotor systems have been linked to altered emotion processing, such as in Parkinson's disease [115], autism spectrum disorder [116], and schizophrenia [6], ccPAS may serve as a novel intervention for enhancing emotion recognition abilities. Future studies should investigate whether repeated ccPAS sessions lead to sustained improvements in social perception and whether individual differences in dispositional empathy or alexithymia predict the extent of ccPAS‐induced benefits.

## Author Contributions

Thomas Quettier: Data curation, formal analysis, visualization. Fausto Caruana: Writing – original draft, writing – review and editing. Cristina Scarpazza: Conceptualization, funding acquisition, writing – review and editing. Sara Borgomaneri: Conceptualization, funding acquisition, methodology, resources, supervision, writing – original draft, writing – review and editing.

## Conflicts of Interest

The authors declare no conflicts interest.
